# Homozygous Mutation of *gsdf* Causes Infertility in Female Nile Tilapia (*Oreochromis niloticus*)

**DOI:** 10.3389/fendo.2022.813320

**Published:** 2022-02-15

**Authors:** Dong-Neng Jiang, You-Xing Peng, Xing-Yong Liu, Umar Farouk Mustapha, Yuan-Qing Huang, Hong-Juan Shi, Ming-Hui Li, Guang-Li Li, De-Shou Wang

**Affiliations:** ^1^ Guangdong Province Famous Fish Reproduction Regulation and Breeding Engineering Technology Research Center, Fisheries College of Guangdong Ocean University, Zhanjiang, China; ^2^ Key Laboratory of Freshwater Fish Reproduction and Development (Ministry of Education), Key Laboratory of Aquatic Science of Chongqing, School of Life Sciences, Southwest University, Chongqing, China

**Keywords:** TGF-β signal pathway, estrogen, CRISPR/Cas9, oogenesis, infertility, Nile tilapia

## Abstract

Gonadal somatic cell-derived factor (Gsdf) is a member of the TGF-β superfamily, which exists mainly in fishes. Homozygous *gsdf* mutations in Japanese medaka and zebrafish resulted in infertile females, and the reasons for their infertility remain unknown. This study presents functional studies of Gsdf in ovary development using CRISPR/Cas9 in Nile tilapia (*Oreochromis niloticus*). The XX wild type (WT) female fish regularly reproduced from 12 months after hatching (mah), while the XX *gsdf* ^-/-^ female fish never reproduced and were infertile. Histological observation showed that at 24 mah, number of phase IV oocyte in the XX *gsdf* ^-/-^ female fish was significantly lower than that of the WT fish, although their gonadosomatic index (GSI) was similar. However, the GSI of the XX *gsdf* ^-/-^ female at 6 mah was higher than that of the WT. The mutated ovaries were hyperplastic with more phase I oocytes. Transcriptome analysis identified 344 and 51 up- and down-regulated genes in mutants compared with the WT ovaries at 6 mah. Some TGF-β signaling genes that are critical for ovary development in fish were differentially expressed. Genes such as *amh* and *amhr2* were up-regulated, while *inhbb* and *acvr2a* were down-regulated in mutant ovaries. The *cyp19a1a*, the key gene for estrogen synthesis, was not differentially expressed. Moreover, the serum 17β-estradiol (E_2_) concentrations between XX *gsdf* ^-/-^ and WT were similar at 6 and 24 mah. Results from real-time PCR and immunofluorescence experiments were similar and validated the transcriptome data. Furthermore, Yeast-two-hybrid assays showed that Gsdf interacts with TGF-β type II receptors (Amhr2 and Bmpr2a). Altogether, these results suggest that Gsdf functions together with TGF-β signaling pathway to control ovary development and fertility. This study contributes to knowledge on the function of Gsdf in fish oogenesis.

## 1 Introduction

Gonadal somatic cell-derived factor (Gsdf) is a secretory protein of the transforming growth factor β (TGF-β) superfamily, which exists in most vertebrates but is lost in some tetropods including mammals (1). First cloned in the rainbow trout (*Oncorhynchus mykiss*) 15 years ago, Gsdf has since been widely studied, especially in teleosts (2). Gsdf is predominantly expressed in the somatic cells of ovaries and testes, but its expression is male-dominant in most fish species (1).

In Philippine medaka (*Oryzias luzonensis*) and sablefish (*Anoplopoma fimbria*), the *gsdfY* that evolved from autosomal *gsdf via* allelic diversification is the male sex determination gene (3, 4). Autosomal *gsdf* is required for testicular differentiation in Japanese medaka (*O. latipes*) and Nile tilapia (*Oreochromis niloticus*), as XY *gsdf* ^-/-^ individuals undergo sex reversal from male to female (5–7). However, the *gsdf* homozygous mutation does not cause the male to female sex reversal in zebrafish (*Danio rerio*), indicating that the Gsdf function in male sex differentiation is not conserved among fish species (8).

In Japanese medaka, *gsdf* is located within an evolutionarily conserved gene cluster containing several genes preferentially expressed in previtellogenic oocytes, indicating the possible Gsdf role in ovary development (9). Most *gsdf* ^-/-^ female Japanese medaka are infertile, but few females are sub-fertile, suggesting that Gsdf is also important for oogenesis (10), yet *gsdf* ^-/-^ female zebrafish are infertile (8). The homozygous mutation of *gsdf* causes ovarian hyperplasia and early-stage oocyte-arrest in zebrafish and Japanese medaka (8, 10). However, the detailed mechanisms of infertility in *gsdf* ^-/-^ female fish and the role of Gsdf in ovary development remain unknown.

Oogenesis involves ovary formation, development and maturation of female gametes, and finally ovulation (11). The hormones secreted by the hypothalamic-pituitary-gonadal (HPG) axis regulate oogenesis in vertebrates, including fish (12–15). Moreover, many genes of the TGF-β signaling pathway expressed in the HPG axis are critical in oogenesis and hormone regulation. The TGF-β signaling pathway consists of several extracellular ligands (phylogenetically divided into TGF-β and BMP groups), cell membrane receptors (types I and II), intracellular Smad proteins, and other functionally related components (16–18). For example, in zebrafish, mutation of three genes from the TGF-β signaling pathway (*amh*, *bmpr1bb* (*alk6b*), and *bmpr2a*) causes ovary hyperplasia and accumulation of immature oocytes (19–21).

In Japanese medaka, the hotei mutation of *amhr2* dysregulates germ cells and arrests follicular development at an early stage (22, 23). Similarly, *amh* and *amhr2* mutations in Nile tilapia cause hypertrophic ovaries with increased primary follicles (24). Serum estradiol (E_2_) levels decrease in the *amh* and *amhr2* female mutants, and E_2_ administration fails to rescue ovary development (24). In mice, Amhr2 is expressed in the brain and pituitary (25). Additionally, in mice and humans, *Amh* directly regulates the secretion of GnRH neurons in the hypothalamus, hence increasing Lh pulsatility and secretion (25). In Nile tilapia, both *amh* and *amhr2* are expressed in the brain and pituitary besides the gonads (24). Decrease of serum E_2_ and down-regulation of the expression of brain *gnrh3*, pituitary *fsh* and *lh*, and ovary *fshr* and *lhr* were observed in *amh* and *amhr2* null Nile tilapia, which was attributed to the down-regulation of the Fsh/Lh signaling (24). Similarly, the expression of *cyp19a1a* in the ovary and E_2_ in the body significantly decreased in the female *gsdf* ^-/-^ zebrafish (8). While the relationship between Gsdf, Fsh/Lh and E_2_ in fish remains unclear, these reproduction-related hormones and the TGF-β signaling genes are attractive enigmas.

Candidate Gsdf receptors are unknown, but Gsdf probably shares known TGF-β receptors with other ligands since TGF-β family members share the limited type I and II receptors. Synteny analysis of zebrafish, spotted gar (*Lepisosteus oculatus*), and humans indicate that *gsdf*, *bmp15*, and *gdf9* were originally paralogs (8). The Gsdf protein might also bind to similar Bmp15 and Gdf9 receptors. As previously mentioned, *amh*, *amhr2*, *bmpr1bb*, and *bmpr2a* mutants cause ovary hyperplasia with immature oocytes, which phenocopy the *gsdf* female mutants in fish (19–24). The phenotype similarity of these TGF-β signaling mutants indicates that these genes might influence each other *via* similar signaling pathways. Moreover, Gsdf might share similar receptors with other TGF-β ligands, but evidence to identify the Gsdf receptors is lacking.

The Nile tilapia is an important farmed fish with an XX-XY sex determination system (26). Gene editing technology has been established in Nile tilapia, and the availability of high-quality genome sequences makes this species an excellent model for studying gene function (27–30). Previously, we established a *gsdf* knockout line in Nile tilapia and illustrated its role in male sex differentiation, while its function in ovary development is still unclear (7). Thus, this study analyzed the XX *gsdf* homozygous mutant fish to determine the gonadal histology, gonadal gene expression and serum steroid hormone levels. In addition, yeast-two-hybrid assays were performed to identify the Gsdf receptors. This study sought to decipher the plausible reasons for the infertility of the female *gsdf* mutants. This study contributes to knowledge on the function of *gsdf* in regulating fish oogenesis.

## 2 Materials and Methods

### 2.1 Animals and Screen of XX *gsdf* Mutants

In the previous study, the Nile tilapia XY *gsdf* F0 mutants were obtained using CRISPR/Cas9 at the Southwest University, China (7). Male and female siblings of heterozygous F1 mutants (*gsdf* ^+/-^) carrying the same mutation (4 bp deletion) were transferred and reared in Guangdong Ocean University, China. The 4 bp deletion occurs at the first *gsdf* exon, which causes a frame-shift mutation, making Gsdf dysfunctional (7).

The Nile tilapia were reared in aerated recirculating freshwater tanks at 26~28°C. XX *gsdf* ^-/-^ and *gsdf* ^+/+^ progenies were obtained by crossing an XX *gsdf* ^+/-^ female with XY *gsdf* ^+/-^ male fish. Genotyping using a sex-linked marker (Marker-5) determined the genetic sex of each fish (31, 32). The *gsdf* mutant fish were detected using the *Bst*NI enzyme, polyacrylamide gels electrophoresis (PAGE), or Sanger sequencing (**Figure S1**). Primer sequences are listed in **Table S1**. The study did not involve endangered or protected species. All animal experiments involving tilapia were performed according to the protocol of the Animal Research and Ethics Committee of Guangdong Ocean University.

### 2.2 Sampling and Histological Analysis

The morphology and histology of XX *gsdf* ^+/+^ and *gsdf* ^-/-^ fish ovaries were analyzed at 6 and 24 months after hatching (mah) to study the role of *gsdf* in ovary development. The body and gonad weight were measured to determine the gonadosomatic index (GSI, gonad weight/body weight). The ovaries of the XX *gsdf* ^+/+^ and *gsdf* ^-/-^ fish were dissected, and one part of each ovary was snap-frozen in liquid nitrogen and stored at −80°C for RNA extraction. The other part was fixed with Bouin’s solution for 24 h at room temperature. The fixed gonads were embedded in paraffin and used for histological analysis. Tissue blocks were sectioned at 5 µm thickness, and the sections were stained with hematoxylin and eosin (HE).

### 2.3 Oocyte Count at Different Phases

The number of oocytes at different development phases in the ovaries of XX *gsdf* ^+/+^ and *gsdf* ^-/-^ fish was compared at 6 and 24 mah. The histological classification of the oocyte phase was performed as previously described (33). Briefly, the oocyte numbers at different phases were counted from photos of the HE-stained sections of the XX *gsdf* ^+/+^ and *gsdf* ^-/-^ ovaries.

### 2.4 Fertility Analysis

XX *gsdf* ^-/-^ and *gsdf ^+^* ^/+^ individuals over 10 mah were cultured in tanks with circulating water at 26°C and fed twice a day (08:00 am and 16:00 pm). The tanks had a layer of crude river sand laid at the bottom for the fish to nest and spawn. Every day from 12:00 to 18:00 hours, when the fish started nesting on the sand, evidenced by their protruding genital papilla, the fish were taken out and squeezed slightly on the abdomen to strip their eggs into a clean bowl. The collected eggs were artificially fertilized using sperms from wild-type XY male fish, and the embryos were hatched at 26~28°C.

### 2.5 Transcriptome Analysis

A transcriptome analysis was performed on the XX *gsdf* ^-/-^ ovaries from fish at 6 mah to understand the causes of delayed oocyte development, infertility of the XX *gsdf* ^-/-^ fish, and the *gsdf* function in ovary development. Eight ovaries were sampled from XX *gsdf* ^+/+^ (n=4) and *gsdf* ^-/-^ (n=4) fish. Total RNA was extracted from the ovaries using the RNeasy Mini Kit (Qiagen, Hilden, Germany), following the manufacturer’s instructions. The mRNA was enriched with a poly-A tail using oligo (dT) coupled to magnetic beads, and the mRNA was randomly interrupted to about 200 bp short fragments.

The first-strand cDNA was synthesized in the M-MuLV reverse transcriptase system (M0253S, NEB, US), and the second-strand was synthesized from dNTPs in the DNA polymerase I system (E7530, NEB, US). These short fragments were ligated to sequencing adapters and sequenced on Illumina Hiseq 2500 platform following the manufacturer’s protocol (Illumina, CA, USA). The raw reads were deposited in the National Center for Biotechnology Information (NCBI) Sequence Read Archive (SRA) database with BioProject number PRJNA703049 (SRR13752974-SRR13752981).

Adapter sequences were removed. The reads containing poly-N (unable to determine base information) and low-quality reads were also removed using fastp (version 0.18.0). Finally, the adapters read with more than 10% unknown nucleotides and over 50% low-quality nucleotides (Q-value ≤ 20) were also removed. Clean reads from each library were aligned to the reference genome using the HISAT2 program (34). The *Oreochromis niloticus* (Orenil1.0) reference genome was downloaded from Ensembl (http://www.ensembl.org/Oreochromis_niloticus/Info/Index). Gene expression levels were estimated using the Fragments Per Kilobase of transcript per Million mapped reads (FPKM) method. The Stringtie assembler (35) reconstructed the transcripts from HISAT2, which were quantified using RSEM (36).

Differential expression analysis was performed using the DESeq2 package (37). Significant differentially expressed genes (DEGs) were identified at |log2(*gsdf* ^-/-^ FPKM/*gsdf* ^+/+^ FPKM)| > 1.0 with < 0.05 false discovery rate (FDR). Gene Ontology (GO)(http://www.geneontology.org/) and Kyoto Encyclopedia of Genes and Genomes (KEGG) enrichment analyses were performed using the significant DEGs. The GO and KEGG enriched terms with P <0.05 were considered significantly enriched.

### 2.6 Real-Time PCR (qPCR)

Ovaries of the XX *gsdf* ^+/+^ and *gsdf* ^-/-^ fish were dissected at 6 mah for qPCR gene expression assays to validate the transcriptome results. Total RNA (1.0 μg) was extracted using the Rneasy Mini Kit (Qiagen, Hilden, Germany) and reverse transcribed to cDNA using the PrimeScript™ RT reagent Kit with gDNA Eraser (Perfect Real Time) (RRO41A, Takara Bio, Kusatsu-Shiga, Japan). Real-time PCR was performed using the SYBR Green qPCR Mix (Dongsheng Biotech, Guangdong, China) following the manufacturer’s instructions on a LightCycler real-time quantitative PCR system (Roche, Basel, Switzerland). The Nile tilapia *β-actin* gene was used to normalize the gene expressions. The relative abundance of the genes was evaluated using the formula R = 2^−ΔΔCt^ (38). Primer sequences used for real-time PCR are listed in **Table S1**.

### 2.7 Immunofluorescence (IF) Analysis

At 6 mah, the ovaries of *gsdf* ^+/+^ and *gsdf* ^-/-^ XX fish were dissected, and the gonads were fixed in Bouin’s solution for 24 hours at room temperature, dehydrated, and embedded in paraffin. Tissue blocks were sectioned at 5 μm thickness, and immunohistochemistry was performed using the Gsdf, Amh and Cyp19a1a antibodies diluted at 1:1000, 1:500 and 1:2000, respectively. Gsdf and Amh antibodies were obtained from previous studies (7, 26), where their specificities were checked. The Cyp19a1a antibody was provided by Professor Yoshitaka Nagahama, National Institute for Basic Biology Okazaki, Japan (39).

For IF, Alexa Fluor 586-conjugated secondary antibodies (Invitrogen, MA, USA) were diluted at 1:500 in blocking solution and incubated with tissue to detect primary antibodies. The nuclei were stained by 4’,6’-diamidine-2-phenylindole-dihydrochloride (DAPI) (Invitrogen, MA, USA). Immunohistochemical analysis was performed as described previously. Photographs were taken under an FV3000 Laser confocal microscope (Olympus, Tokyo, Japan). Finally, the positive signals were quantified using Image J software (National Institute of Health, MD, USA) following the instructions provided.

### 2.8 Measurement of Serum Steroid Hormones

The influence of *gsdf* mutation on the serum steroid hormone levels was analyzed. Blood samples were collected from the caudal vein of XX *gsdf* ^+/+^ and *gsdf* ^-/-^ fish using syringes at 6 and 24 mah. The blood samples were stored at 4°C overnight and centrifuged at 5,000 g for 5 min at 4°C. The upper layer serum was collected and stored at -80°C until use. The serum 17β-estradiol (E_2_), 11-keto Testosterone (11-KT), Testosterone (T), and Pregnenolone (P5) levels were measured using Enzyme Immunoassay (EIA) Kits (E_2_, 11-KT and T kits from Cayman Chemical Company (MI, USA), and the P5 kit from Abnova (Taipei, China) following the manufacturer’s instructions.

### 2.9 Yeast-Two-Hybrid Assays

Yeast-two-hybrid assays were performed to identify the possible Gsdf receptor. Firstly, the *gsdf* was constructed into the pBT3-SUC bait vector. Concurrently, the *amhr2* and *bmpr2a* were constructed into the pPR3 prey vector. Next, the bait plasmid + pPR3-N, bait and prey plasmid, bait plasmid + POST-Nubal, pTSU2-APP+pNubG-Fe65 (positive control), and pTSU2-APP+pPR3-N (negative control) were co-transformed into NMY51 yeast strain. The bait plasmids + pPR3-N functioned as autoactivation assays, and bait plasmids + POST-Nubal functionally validated the ubiquitin system. After that, yeasts were cultured in DDO (Lack of LEU and TRP) liquid medium to the optical density (OD600) of ~0.4-0.8. Cultures were titrated 1:10, 1:100, 1:1,000, and 1:10,000 in saline solution.

Then, 10 μl of each dilution was seeded on DDO, TDO (Lack of LEU, TRP and HIS) and QDO (Lack of LEU, TRP, HIS and ADE) plates. The seeded plates were incubated at 30°C for 72 h to observe the yeast growth. Yeast growth assays estimated the protein-protein interaction strength on plates lacking HIS (suitable for weak protein-protein interactions) or ADE (suitable for strong protein-protein interactions).

### 2.10 Statistical Analysis

All data are presented as mean ± SD from at least three independent samples collected from different fish, and Student’s t-test analyzed the statistical significance. The significance level was *P* < 0.05, and statistical tests were performed with the Statistical Package for Social Sciences (SPSS) 19.0 (IL, USA).

## 3 Results

### 3.1 *gsdf* Homozygous Mutation Causes Infertility in Female Nile Tilapia

**Figure 1:**
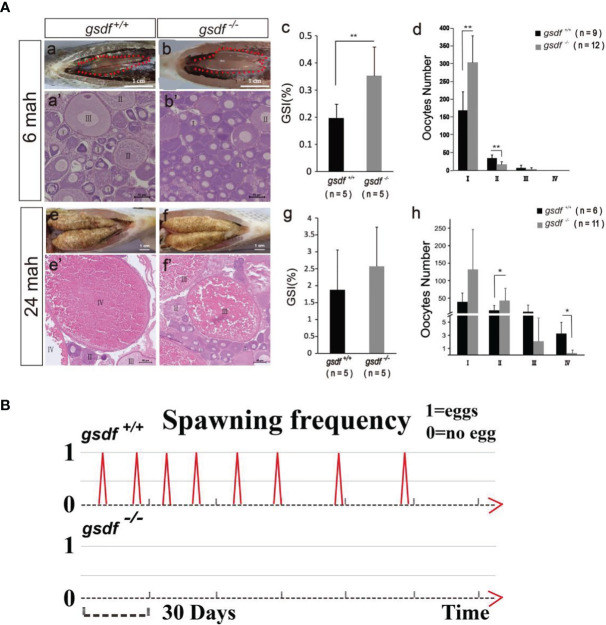
Morphological and histological analyses of ovaries **(A)** and spawning frequency **(B)** from XX *gsdf* ^+/+^ and *gsdf ^-^* ^/-^ tilapia. The fish were dissected at 6 and 24 mah. Results are presented as mean ± SD. * and ** indicate significant differences at *P* < 0.05 and *P* < 0.01, respectively. **(B)** The spawning frequency of 12-months old XX *gsdf* ^+/+^ and *gsdf* ^-/-^ fish were observed for half a year. More than ten XX *gsdf* ^-/-^ fish were observed for spawning, while none reproduced.

At 6 and 24 mah, there was no observable morphological difference between XX *gsdf* ^+/+^ and *gsdf* ^-/-^ fish (**Figure S2**). The GSI of XX *gsdf* ^-/-^ was significantly higher than that of XX *gsdf* ^+/+^ fish at 6 mah (**Figures 1A, a–c**, **Table S2**). The number of phase I oocytes in the XX *gsdf* ^+/+^ ovaries was significantly lower than that of XX *gsdf* ^-/-^ ovaries at 6 mah. Nevertheless, the number of phase II oocytes in the XX *gsdf* ^+/+^ fish was substantially higher than that of the XX *gsdf* ^-/-^ fish (**Figures 1A, a’, b’, d**). These results indicate that the ovaries of XX *gsdf* ^-/-^ fish were hyperplastic, and the oocytes were arrested at the primary growth stage.

There were many maturing oocytes in XX *gsdf* ^+/+^ fish ovaries at 24 mah, while the ovaries of XX *gsdf* ^-/-^ fish had very few mature and many white dead oocytes (**Figures 1A, e-f**). Mutant gonads contained less-maturing oocytes, but the GSI between XX *gsdf* ^+/+^ and *gsdf* ^-/-^ fish at 24 mah were similar, indicating that the mutant gonads were still hyperplastic with more immature oocytes (**Figure 1A, g**). The HE staining showed that XX *gsdf* ^-/-^ ovaries had more phase II oocytes than XX *gsdf* ^+/+^ ovaries at 24 mah. However, the number of phase IV oocytes in the XX *gsdf* ^-/-^ ovaries was significantly lower than that of the XX *gsdf* ^+/+^ ovaries (**Figures 1A, e’-f’, h**). These results indicate that the development of oocytes in XX *gsdf* ^-/-^ fish ovaries was delayed and immature at 24 mah, corresponding to the previtellogenic phase.

The XX *gsdf* ^+/+^ fish became sexually mature at approximately one year and spawned eggs continuously, with the shortest interval at 14 days. Additionally, the spawning, fertilization, and hatching are normal, with normally-developed fry. However, the XX *gsdf* ^-/-^ fish (n=10) showed no spawning, indicating their sterility (**Figure 1B**).

### 3.2 Loss of *gsdf* Alters Gene Expression in the Ovaries of XX *gsdf* ^-/-^ Fish

The histology difference in the ovaries of XX *gsdf* ^+/+^ and *gsdf* ^-/-^ fish are observably different at 24 mah. Thus, the ovaries of the 6-months old fish were chosen for gene expression analysis to identify the effect of *gsdf* mutation on gene expressions.

A quality control analysis of raw sequences using the fastp software filtered out low quality reads, resulting in 22.96 and 22.38 Gb of cleaned transcriptome data from the ovaries of XX *gsdf* ^+/+^ and *gsdf* ^-/-^ fish, respectively. The average Q20 and Q30 of the clean data were 97.80% and 93.65%, respectively. An average of 95.6% and 94.7% clean reads from XX *gsdf* ^+/+^ and *gsdf* ^-/-^ fish were mapped to the reference genome. The sequencing and mapping statistics for the eight transcriptomes are listed in **Table S3**. There were 13,133, and 13,722 genes (FPKM > 1) expressed in the ovaries of the XX *gsdf* ^+/+^ and *gsdf* ^-/-^ fish, receptively. A total of 395 DEGs were identified in XX *gsdf* ^+/+^ and *gsdf* ^-/-^ ovaries at 6 mah (FDR <0.05). The ovaries of XX *gsdf* ^-/-^ fish had 344 and 51 up-regulated and down-regulated genes, respectively (**Table S4**).

**Figure 2:**
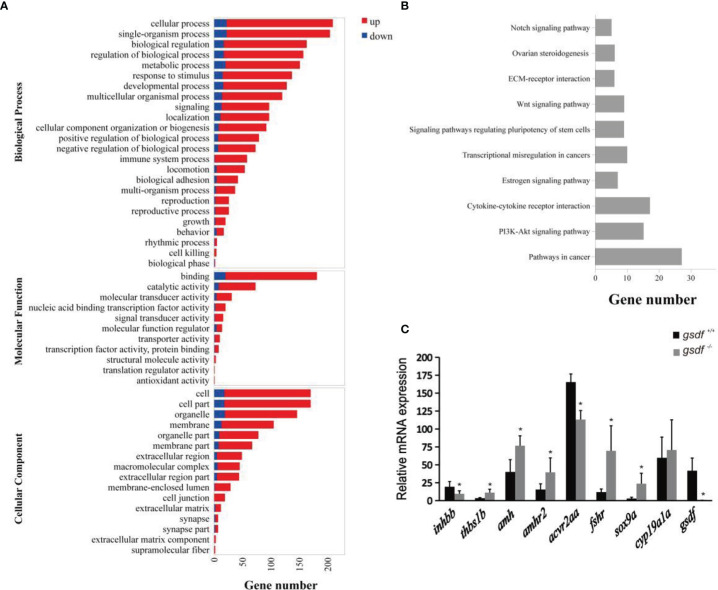
Transcriptome analysis of gene expression in ovaries of XX *gsdf* ^-/-^ and *gsdf* ^+/+^ tilapia at 6 mah. **(A)** GO enrichment analysis of DEGs. Red and blue indicate up-regulated and down-regulated DEGs, respectively. **(B)** The top ten enriched KEGG pathways of the DEGs. **(C)** qPCR validation of the transcriptome data. All the examined genes showed similar expression patterns with the transcriptome data. Results are presented as mean ± SD. * indicates significant difference at *P* < 0.05.

The DEGs enriched GO terms at the three main functional categories, including biological process (24 terms), molecular function (11 terms), and cellular component (16 terms) (**Figure 2A**). Under biological process, the DEGs significantly enriched cellular process (GO:0009987, 209 genes) (187 genes up-regulated and 22 genes down-regulated), single-organism process (GO:0044699, 204 genes) (182 genes up-regulated and 22 genes down-regulated), and biological regulation (GO:0065007, 163 genes) (146 genes up-regulated and 17 genes down-regulated) terms. Within the molecular function, the DEGs enriched binding (GO:0005488, 181 genes) (161 genes up-regulated and 20 genes down-regulated) and catalytic activity (65 genes up-regulated and eight genes down-regulated) (GO:0003824, 73 genes). In the cellular component, the DEGs were significantly enriched in cell (GO:0005623, 170 genes) (152 genes up-regulated and 18 genes down-regulated), cell part (GO:0044464, 170 genes) (152 genes up-regulated and 18 genes down-regulated) and organelle (GO:0043226, 146 genes) (127 genes up-regulated and 19 genes down-regulated) GO term (**Figure 2A**). The DEGs that enriched these GO terms are listed in **Table S5**.

The KEGG enrichment analysis showed that 178 DEGs enriched 244 signaling pathways. The top four enriched KEGG pathways include cancer, cytokine-cytokine receptor interaction, ovarian steroidogenesis, and estrogen signaling pathway (**Figure 2B** and **Table S6**). In the steroidogenesis pathway, two genes, *fshr* and *hsd17b1*, were significantly up-regulated in mutant fish. Several DEGs were enriched in the TGF-β signaling pathway, including *inhbb*, *acvr2aa*, *thbs1b, amh* and *amhr2.* In addition, qPCR confirmed that *gsdf*, *inhbb* and *acvr2aa* were significantly down-regulated, while *thbs1b, amh, amhr2, fshr*, and *sox9a* were up-regulated considerably in *gsdf* ^-/-^ ovaries, verifying the gonadal transcriptomic data (**Figure 2C**).

### 3.3 Immunofluorescence of Gsdf, Cyp19a1a and Amh Expression in the Ovaries of XX *gsdf* ^-/-^ Fish at 6 mah

**Figure 3:**
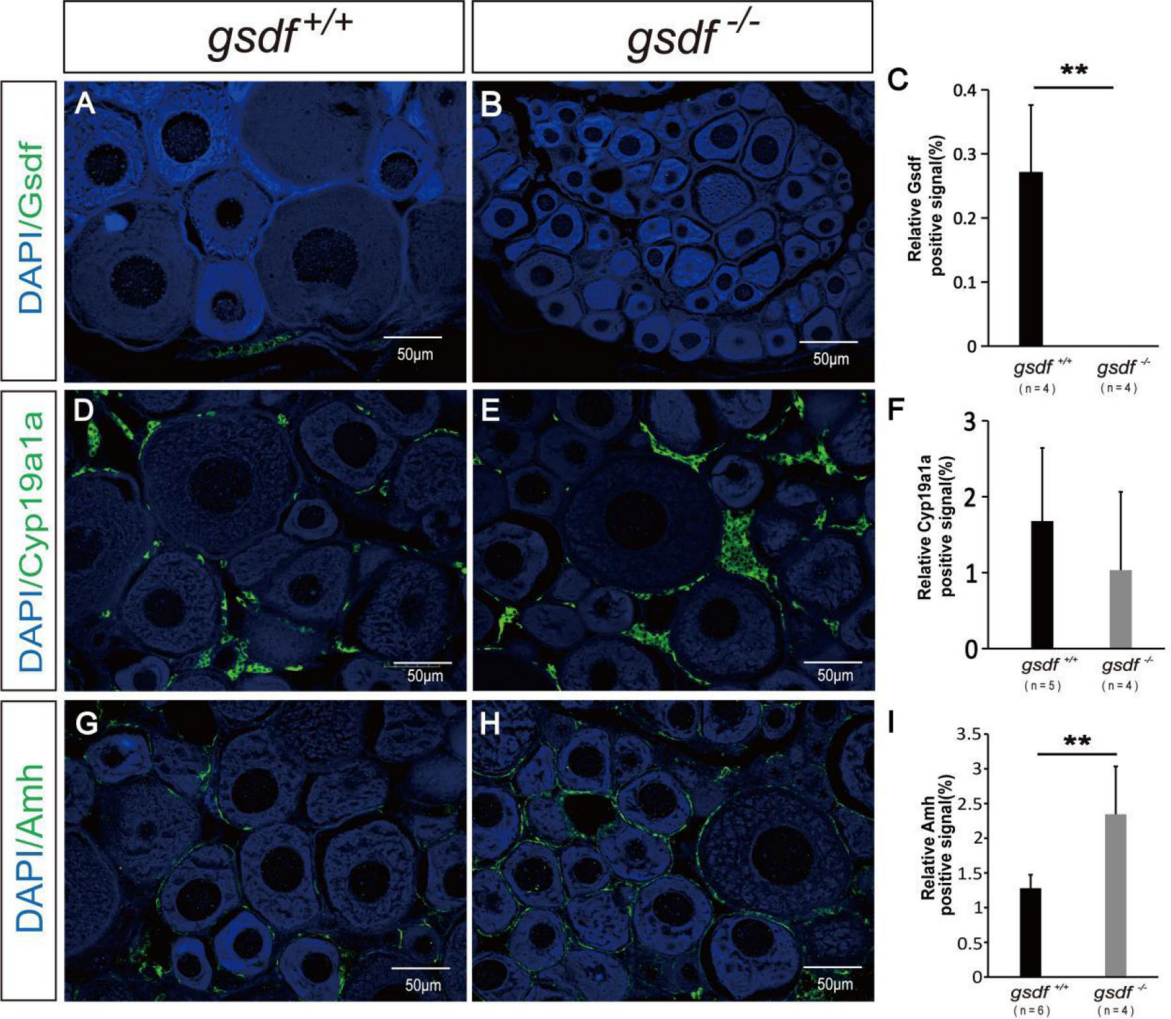
Immunofluorescence analysis of Gsdf, Cyp19a1a and Amh in ovaries of XX *gsdf* ^-/-^ and *gsdf* ^+/+^ tilapia at 6 mah. Gsdf **(A, B)**, Cyp19a1a **(D, E)** and Amh **(G, H)**. Green fluorescence represents positive signal. Blue fluorescence represents the DAPI signal. The statistical analysis of the positive signal intensity **(C, F, I)**. Results are presented as mean ± SD. ** indicates significant difference at *P* < 0.01.

Firstly, the expression of Gsdf positive signal was detected in the ovaries of XX *gsdf* ^+/+^ fish. While the Gsdf signal was absent in the XX *gsdf* ^-/-^ fish ovaries at 6 mah, confirming that the Gsdf protein was wholly removed from the XX *gsdf* knockout fish ovary (**Figures 3A**–**C**). Estradiol (E_2_) is critical for fish ovary development, and *cyp19a1a* encodes aromatase, the rate-limited enzyme for E_2_ synthesis. The gonad transcriptome and qPCR analyses showed that the expression of *cyp19a1a* in *gsdf* ^-/-^ and wild-type fish are indistinguishable. Therefore, an IF analysis was performed to confirm the protein level expression of Cyp19a1a. The Cyp19a1a signal was present in the interstitial and follicle cells of the ovaries from both XX *gsdf* ^+/+^ and *gsdf* ^-/-^ fish. The fluorescence signal intensity between the knockout and wild-type ovaries was similar (**Figures 3D**–**F**). Amh positive signals were also observed in the follicle cells of the ovaries of both XX *gsdf* ^+/+^ and *gsdf* ^-/-^ fish at 6 mah (**Figures 3G, H**). Moreover, the quantitative Amh positive signal in the XX *gsdf* ^-/-^ ovary was significantly higher than the XX *gsdf* ^+/+^ ovary at 6 mah, consistent with the qPCR results (**Figure 3I**).

### 3.4 The Serum Steroid Hormones Level in XX *gsdf* ^+/+^ and *gsdf ^-^* ^/-^ Fish

**Figure 4:**
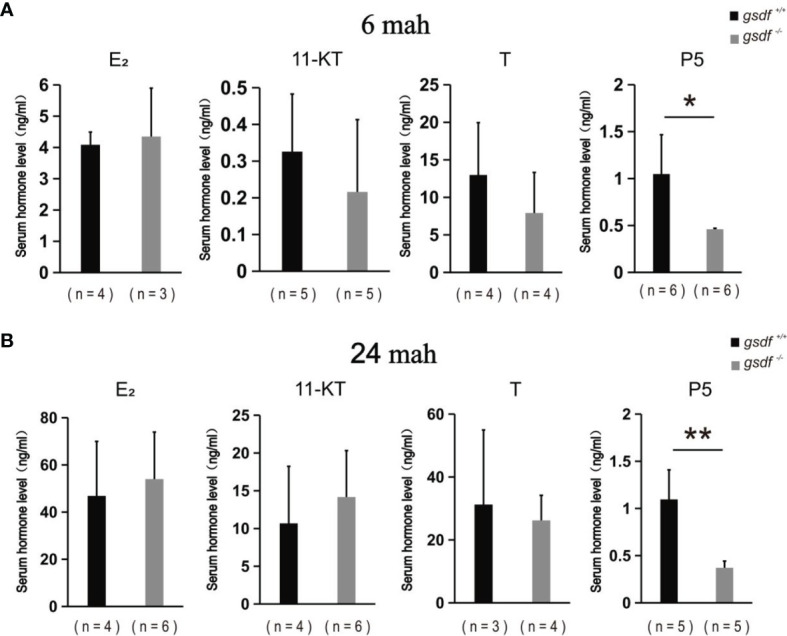
Serum E_2,_ 11-KT, T and P5 levels of XX *gsdf* ^+/+^ and *gsdf ^-^* ^/-^ tilapia at 6 **(A)** and 24 **(B)** mah. Results are presented as mean ± SD. * and ** above the error bar indicate significant difference at *P* < 0.05 and *P* < 0.01, respectively.

The serum steroid hormone concentration was examined in the XX *gsdf* ^+/+^ and *gsdf* ^-/-^ fish at 6 and 24 mah to detect whether steroid hormones affect female sterility. There was no significant difference in E_2_, 11-KT and T levels in sera from XX *gsdf* ^+/+^ and *gsdf* ^-/-^ fish at both 6 and 24 mah (**Figures 4A, B**). However, the P level in XX *gsdf* ^+/+^ was significantly higher than *gsdf* ^-/-^ fish at both 6 and 24 mah.

### 3.5 Characterization of Gsdf Receptors

**Figure 5:**
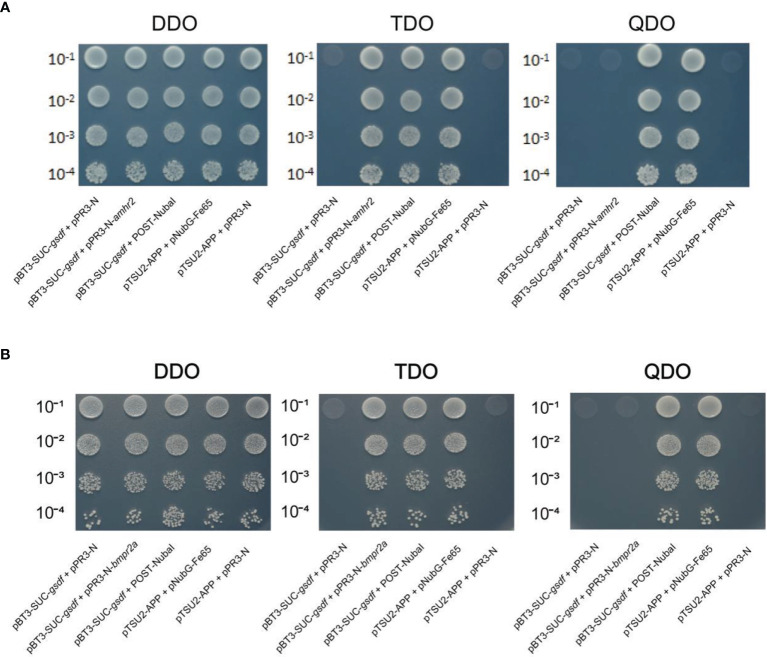
Yeast-two-hybrid assays for Gsdf (bait) interaction with the prey [Amhr2 **(A)** and Bmpr2a **(B)**] proteins. The hybridized yeast cell that survived on TDO and QDO plates indicate weak and strong positive interactions, respectively. The pBT3-SUC-*gsdf* + pPR3-N was set for detection of self-activation, while pBT3-SUC-*gsdf* + POST-Nubal was performed for functional verification of the ubiquitin system. The pTSU2-APP + pNubG-Fe65 and pTSU2-APP + pPR3-N were used as the positive and negative control, respectively.

Yeast-two-hybrid assays verified the interaction between Gsdf and Amhr2, and Gsdf and Bmpr2a. NMY51 yeast co-transformed with the selected plasmids grew on the DDO plate, indicating successful transformation of the plasmids into yeast. The yeast co-transformed with the negative control plasmids did not grow on TDO and QDO plates, indicating that they did not self-activate the expression of *HIS3* and *ADE2* reporter genes, respectively (**Figure 5**). Whereas the yeast co-transformed with the positive control plasmids grew on the TDO and QDO plates. In addition, the yeast co-transformed with pBT3-SUC-*gsdf* + Post-Nubal plasmids grew on TDO and QDO plates, indicating the functional role of the ubiquitin system. The yeast co-transformed with pBT3-SUC-*gsdf* + pPR3-N did not grow on TDO plates, while the yeast co-transformed with pBT3-SUC-*gsdf* + pPR3-N-*amhr2*, and pBT3-SUC-*gsdf* + pPR3-N-*bmpr2a* plasmids grew on TDO plates, indicating the successful co-transformation and activation of the *HIS3* reporter gene. However, the yeast co-transformed with pBT3-SUC-*gsdf* + pPR3-N-*amhr2*, and pBT3-SUC-*gsdf* + pPR3-N-*bmpr2a* plasmids did not grow on QDO plates, implying that the *ADE2* reporter gene was not activated. These results showed a weak interaction between Gsdf and Amhr2, and Gsdf and Bmpr2a.

## 4 Discussion

The Gsdf protein has remained a research focus since its first discovery in rainbow trout 15 years ago (1, 2). The Gsdf protein is mainly expressed in somatic cells of gonads, and *gsdf* functions in testicular differentiation in Japanese medaka and Nile tilapia (5–7). Loss of *gsdf* caused infertility in female zebrafish and Japanese medaka, indicating that Gsdf is also essential for ovary development in fish. This study analyzed the phenotype of *gsdf* mutant and the possible role of *gsdf* in ovary development in female Nile tilapia.

### 4.1 Phenotypes of *gsdf* Mutants in Fish

In Japanese medaka, the ovaries of the *gsdf* female mutant accumulate numerous small and previtellogenic oocytes while lacking the mature vitellogenic oocytes resulting in low fertility or infertility (10). In zebrafish, most *gsdf* mutant females lay a few eggs at 4.5 mpf (month post-fertilization) but become sterile at 18 mpf (8). In zebrafish, mutant ovaries have more younger oocytes than the wild type. Mutant ovaries at 18 mpf lack mature oocytes, indicating that *gsdf* is required for oocyte maturation and inhibits the accumulation of young ovarian follicles in zebrafish. Similarly, the *gsdf* homozygous mutated ovaries of Nile tilapia had an increased number of primary oocytes at 24 mah, lacked mature oocytes and finally became infertile. Gene knockout study in two Perciformes (Japanese medaka and Nile tilapia) and one Cypriniforme fish (zebrafish) species concluded that *gsdf* is functionally conserved for regulating young oocyte maturity and is critical for fertility in female fish (8, 10). In Nile tilapia, Gsdf is mainly expressed in the somatic cell around the oogonia (7, 40). Gsdf may act as a paracrine factor and directly control the proper proliferation of the oogonia, or control the transition of the oogonia into the oocyte. Gsdf may also act as an autocrine factor and could indirectly influence the oogonia and oocyte *via* somatic cell and germ cell interaction. It is interesting to reveal how Gsdf is involved in oocyte development. In doing so, it is necessary to identify its receptors and where they are localized.

### 4.2 Receptors of Gsdf in Nile Tilapia

Genomics synteny analysis showed that *gsdf* is a paralog of the BMP members (8). Consistently, the yeast-two-hybrid results from this study showed that the Nile tilapia Gsdf binds to type II receptor in BMP signal pathway *in vitro*. Our experimental results confirmed that Gsdf belongs to the BMP group, and it shares the type II receptors with other BMP ligands, which was further proved by gene mutation analysis of TGF-β ligands and receptors.

Similar phenotypes of *gsdf* mutation were observed in other TGF-β ligand/receptor mutants in fish species. In Japanese medaka, the homozygous *hotei* mutation of *amhr2* causes early-stage follicle arrest, the mutant ovaries become hypertrophic, and most mutant female fish become sterile (22). In zebrafish, young *amh* mutants lay very few eggs, while older mutants become infertile with accumulated non-vitellogenic follicles in exceedingly large ovaries (19). *Amhr2* was evolutionarily lost in zebrafish; thus, Amh binds with another type II receptor of the TGF-β signaling pathway (41). Knockouts of *bmpr2a* phenocopied the phenotypes of *amh* mutants in male and female zebrafish, including gonadal hypertrophy, hyperproliferation of germ cells, and retarded gametogenesis. Thus, Bmpr2a is considered a possible type II receptor for Amh in zebrafish (21). Besides, the XX Nile tilapia with *amh* or *amhr2* deficiency displays hypertrophic ovaries with many primary oocytes but fails to reproduce (24).

Both *gsdf* and *amh* are expressed in the granulosa cells of the fish ovary, and both act as paracrine factors which might influence the neighboring germ cells (7, 24). In Nile tilapia, the *amhr2* is expressed in granulosa cells, oogonia and phase I oocytes, indicating that *amh* regulates germ cells directly *via* its type II receptor, Amhr2. This is also supported by the similar phenotypes observed in *amh* and *amhr2* null female Nile tilapia. However, there is still no direct evidence showing that fish Amh binds with Amhr2, as it is with mammals (24). Herein, yeast-two-hybrid experiments showed that Gsdf binds with Amhr2. Therefore, Gsdf might control germ cell development *via* interactions with Amhr2 in Nile tilapia. The oocytes are unable to grow into stage IV either in the *amh* or *amhr2* null ovaries at the adult stage (24), while stage IV oocyte was found in *gsdf* null ovaries in the present study. The similar *amh* and *amhr2* null female phenotype suggests that Amh, instead of Gsdf, probably is the main ligand of Amhr2 in Nile tilapia. Considering that *gsdf*, *amh*, *amhr2*, and *bmpr2a* showed similar phenotypes of ovary development in different fish species, we deduced that *gsdf* and *amh* share similar receptors and control similar downstream genes in different fish species. Their downstream genes control germ cell proliferation and differentiation in the ovary.

### 4.3 Possible Mechanisms of Gsdf Signal in Regulation of Ovary Development

#### 4.3.1 The Influence of Gsdf on Steroid Hormone Synthesis in the Ovary

Estrogens synthesized in somatic and germ cells are critical for normal folliculogenesis of fish ovaries (42, 43). In Nile tilapia, mutations of *amh*, *amhr2*, *foxh1*, and *tsp1a* down-regulate *cyp19a1a* expression in the ovary and serum E_2_ levels (24, 44, 45). The decreased E_2_ levels caused abnormal oocyte development in the mutants. However, exogenous E_2_ supplementation in *amh* and *amhr2* female mutants failed to rescue the folliculogenesis arrest caused by apoptosis of follicle cells (24).

E_2_ levels in the whole body of *gsdf* ^-/-^ female zebrafish were lower than those of the wild-type fish at 11 and 20 mpf (19). The ovary *cyp19a1a* expression also significantly decreased in the *gsdf* ^-/-^ female zebrafish. In the current study, both ovary *cyp19a1a* and serum E_2_ levels of *gsdf* mutants were similar to the wild-type fish at 6 and 24 mah, indicating that Gsdf does not directly control estrogen synthesis in the ovary. The early-stage arrest of oocyte development and infertility of female *gsdf* ^-/-^ Nile tilapia may not be due to either deficiency or excessive estrogen. The estrogens interact with three receptors (Esr1, Esr2a, Esr2b) in Nile tilapia. Functional studies demonstrated that *esr2a* mutants show delayed ovary development (13). In contrast, the transcriptome data showed that none of these *esrs*`s expressions were altered in the *gsdf* mutants. However, we could not exclude the possibility that other molecular components in the estrogen signal pathway in the female *gsdf* ^-/-^ Nile tilapia were affected. Further studies should elucidate the underlying mechanism of *gsdf* mutation on the estrogen signaling pathway, which is critical for the regulation of oogenesis in different fish species.

Moreover, the serum pregnenolone levels significantly decreased in female *gsdf* ^-/-^ than the wild-type fish. The initial step of pregnenolone synthesis involves cholesterol transport to the inner mitochondrial membrane by steroidogenic acute regulatory protein (StAR). StAR is encoded by two paralogous genes, *StAR1* and *StAR2*, of teleosts fish, including Nile tilapia (46). The cholesterol side-chain cleavage enzyme, coded by *P450scc* (*Cyp11a1*), converts cholesterol to pregnenolone (47). The present transcriptome results showed similar expression levels of *StAR1*, *StAR2* and *cyp11a1* between *gsdf* ^-/-^ and wild-type ovaries. Therefore, the decreasing serum pregnenolone levels of *gsdf* ^-/-^ fish are not attributable to these genes. However, protein-level expression of these genes is required to confirm these speculations. Additionally, pregnenolone is the intermediate product for most of steroids, including estrogen. Its decrease might change the activities of downstream enzymes. The decreased E_2_ level in *gsdf* ^-/-^ zebrafish and pregnenolone level in *gsdf* ^-/-^ Nile tilapia indicate that Gsdf controls ovary development by regulating certain steroid hormones in fish. Nevertheless, future studies should address the Gsdf regulatory mechanisms on steroid hormone synthesis in the different fish.

#### 4.3.2 The Influence of Gsdf on Gonadotropin Synthesis

Sex steroid hormone synthesis, gonadal growth, and differentiation are regulated by gonadotropin signals through gonadal G-protein-coupled receptors (48). The gonadotropins include Fsh and Lh, and their mammalian receptors are Fshr and Lhcgr, respectively. Unlike in mammals, both Fsh and Lh interact with Fshr in zebrafish (49). Moreover, disrupting the *fshr* gene causes the arrest of ovarian follicles at the PG-PV transition. Later on, *fshr* is indispensable for folliculogenesis in zebrafish, while *lhcgr* may not be essential for female zebrafish reproduction, indicating that Fshr is more important for normal ovary development than Lhcgr in fish (49).

Besides, Fsh-deficient (*fshb^-/-^*) female zebrafish show delayed puberty onset but fertile, while Lh-deficient (*lhb^-/-^*) females show normal gonadal growth but are infertile from the failure to spawn (50). Pituitary *lhb*, ovary *lhcgr* and *fshr* decrease significantly in female *gsdf* ^-/-^ zebrafish (8). Similarly, pituitary *lhb* and *fsh*, and ovary *lhr* and *fshr* reduced considerably in female *amh* ^-/-^ and *amhr2* ^-/-^ Nile tilapia (24). The decreased gonadotropin signals might be caused by early-stage follicle arrest in the *amh* or *amhr2* mutant fish. The pituitary *fshb* was significantly down-regulated, and ovary *fshr* was significantly up-regulated in female *gsdf* ^-/-^ Nile tilapia (**Figure S3**). Gonadotropin signaling probably decreased in female *gsdf* ^-/-^ Nile tilapia, causing follicle arrest in the mutants. Altogether, *gsdf* possibly regulates ovary development *via* the gonadotropin signaling pathway in fish. However, besides gonads, *amh* and *amhr2* are also expressed in the brain and pituitary of Nile tilapia, while *gsdf* is only expressed in the gonads (24, 40). Consequently, Gsdf might influence the pituitary gonadotropin signaling directly *via* its putative receptors (Amhr2 and Bmpr2a) if it could be transported to the pituitary or brain regions *via* the blood. Further studies are needed to support this speculation.

More evidence proves that the TGF-β signaling genes, besides *Amh*, *gsdf* and *Amhr2* discussed above, are critical for regulating gonadotropin signaling pathways in vertebrates. The other two TGF-β subfamily members, Activin and Inhibin, antagonize each other to regulate pituitary *fshb* expression and Fsh secretion in vertebrates (51). Inhibin contains a unique inhibin-specific α subunit (Inha) and shares a common β subunit (Inhba and Inhbb) with Activin to form Inhibin A, or Inhibin B. Activins are homodimers of either Inhba or Inhbb or their heterodimers. In mammals, Activin stimulates, and Inhibin inhibits the Fsh section (51, 52).

Pituitary expression of *fshb* significantly increases in *inha* ^-/-^ female zebrafish compared with wild-type fish (52). However, the expression of ovarian *inha*, *fshr*, and *lhr* decreases significantly in female *gsdf* ^-/-^ Japanese medaka (10). Previously, IHC analysis showed positive Fshb and Lhb signals in cells of XY *gsdf*-null pituitaries compared with the wild-type males or females, indicating an increased Fsh and Lh secretion in medaka (53). In this study, ovary *inhbb* and pituitary *fshb* genes were significantly down-regulated in female *gsdf* ^-/-^ Nile tilapia. Altogether, *gsdf* mutations possibly influence pituitary *fsh* gene expression in fish *via* the Activin and Inhibin signaling pathway.

#### 4.3.3 The Disorganized Gene Expression in gsdf Mutants

*Gsdf* mutation influences the same genes in different ways in different species, as mentioned above. We recognize that the TGF-β signaling pathway shares the same downstream proteins, such as Smad4, which combine with other Smads to form activated Smad complexes that move to the nucleus to stimulate or inhibit downstream genes. Therefore, these TGF-β ligands are tightly linked to complex gene regulatory networks. Nevertheless, absence of any TGF-β ligand negatively influences the whole TGF-β signaling pathway.

Furthermore, the numbers of TGF-β ligands vary in different species because of gene duplication or loss (16). Thus, deficient of a TGF-β ligand in one species substantially influences the species physiology and homeostasis. However, this phenomenon would improve our understanding of TGF-β ligand mutations in different species and their effects on gene expression. Although *gsdf* mutations exert differential influences on different genes in female fish from different species. The final, infertile phenotype of female mutants was consistent and conserved in three unrelated fish species (Japanese medaka, zebrafish, and Nile tilapia). The infertility of *gsdf* mutants is probably due to genetic toxicity caused by the disorganized TGF-β signaling in the ovary after mutation. The KEGG analysis enriched more than 20 genes in the cancer pathway and supported our speculation that the *gsdf* mutated fish is unhealthy and has a defect in the ovary. The *gsdf* mutated female fish could be used as an ovary development disorder model caused by TGF-β signaling deficiency in vertebrates.

## 5 Conclusion

This study demonstrated that female *gsdf* ^-/-^ Nile tilapia was infertile, and the development of oocytes was arrested at an early stage. There was no significant changes in serum estrogen level and ovarian *cyp19a1a* expression in *gsdf* knockout fish, indicating that infertility in *gsdf* mutants was not caused by estrogen deficiency. The expression of several TGF-β signaling genes changed significantly in the ovaries of *gsdf* knockout fish, which may be the reason for its infertility. In addition, two possible type II receptors of Gsdf were identified by yeast-two-hybrid assays for the first time. These results enrich our understanding of the role of Gsdf in the oogenesis of teleosts.

## Data Availability Statement

The datasets presented in this study can be found in online repositories. The names of the repository/repositories and accession number(s) can be found below: https://www.ncbi.nlm.nih.gov/, SRR13752974-SRR13752981.

## Ethics Statement

The animal study was reviewed and approved by Animal Research and Ethics Committee of Guangdong Ocean University.

## Author Contributions

D-NJ and Y-XP took part in the whole process of the experiment, and wrote the draft of this manuscript. X-YL, Y-QH, and UM participated in the experiments. M-HL, H-JS, and G-LL co-conceived the experiment. UM revised the draft. D-SW and D-NJ designed and supervised the experiments. All authors read and approved the final manuscript. All authors approved the final version to be published.

## Funding

The authors thank the financial support provided by the Key Project of “Blue Granary Science and Technology Innovation” of the Ministry of Science and Technology (2018YFD0900202); the National Natural Science Foundation of China (Project Nos. 31702326, 32002367, 32072960 and 32172971).

## Conflict of Interest

The authors declare that the research was conducted in the absence of any commercial or financial relationships that could be construed as a potential conflict of interest.

## Publisher’s Note

All claims expressed in this article are solely those of the authors and do not necessarily represent those of their affiliated organizations, or those of the publisher, the editors and the reviewers. Any product that may be evaluated in this article, or claim that may be made by its manufacturer, is not guaranteed or endorsed by the publisher.
