# Supplementary material for: Homozygous Mutation of gsdf Causes Infertility in Female Nile Tilapia (Oreochromis niloticus)
Source: Front Endocrinol (Lausanne). 2022 Feb 15;13:813320. doi: 10.3389/fendo.2022.813320 (PMC8886716; doi:10.3389/fendo.2022.813320)
Supplement: Supplementary file 2 [file DataSheet_2.docx]

**Supplementary Figures**


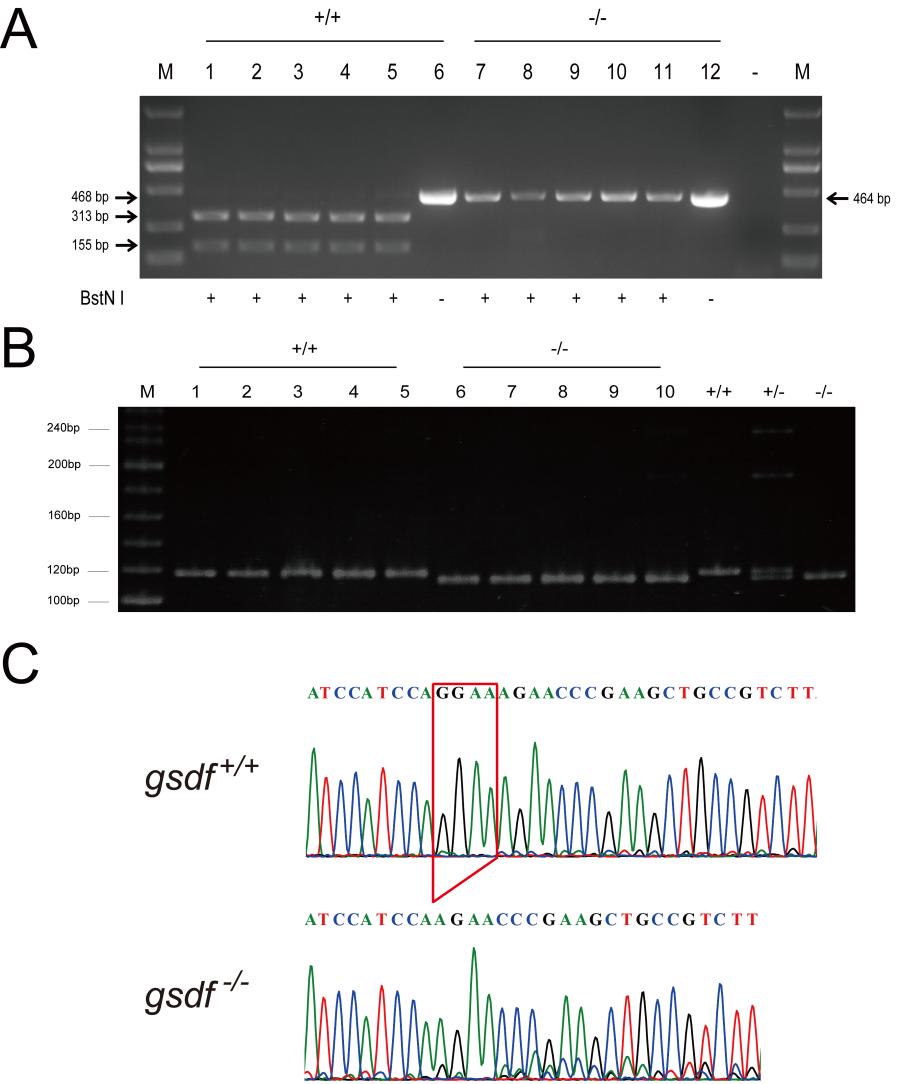


**Supplementary Figure 1**. Genotyping of *gsdf* mutants. (A) Detection of XX *gsdf* ^+/+^ and *gsdf* ^-/-^ fish by restriction enzyme digestion (BstNI, NEB). (B) F2 genotypes identified by heteroduplex motility assay. (C) Sanger sequencing confirmed that XX *gsdf* ^-/-^ fish lost 4 bp. M, DNA marker.


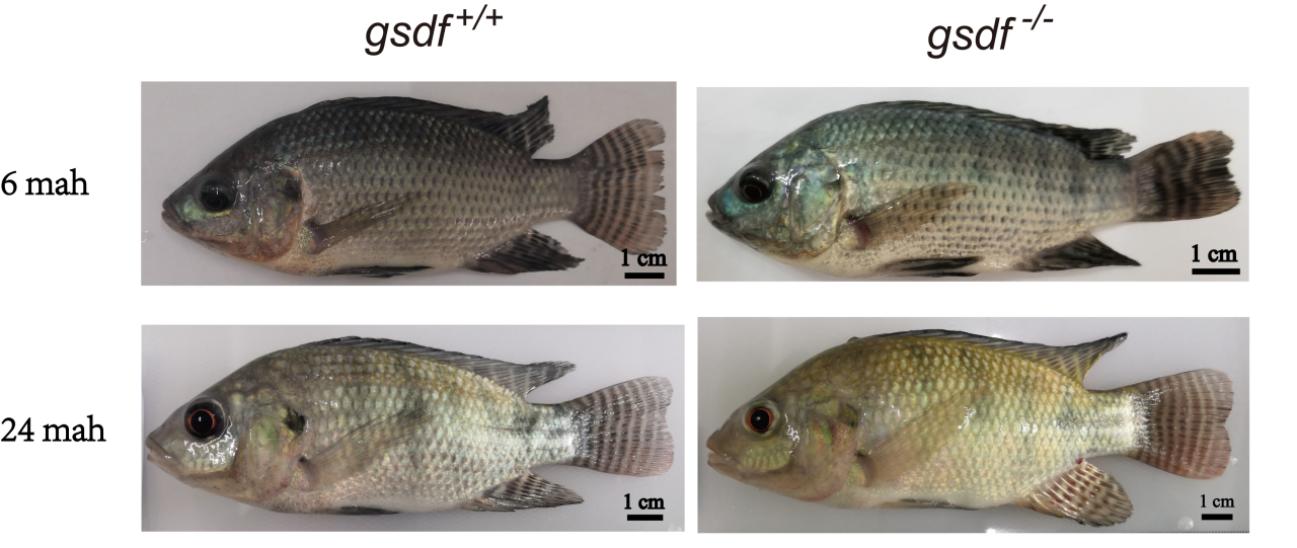


**Supplementary Figure 2**. Morphological characteristics of the XX *gsdf* ^-/-^ and *gsdf* ^+/+^ fish.


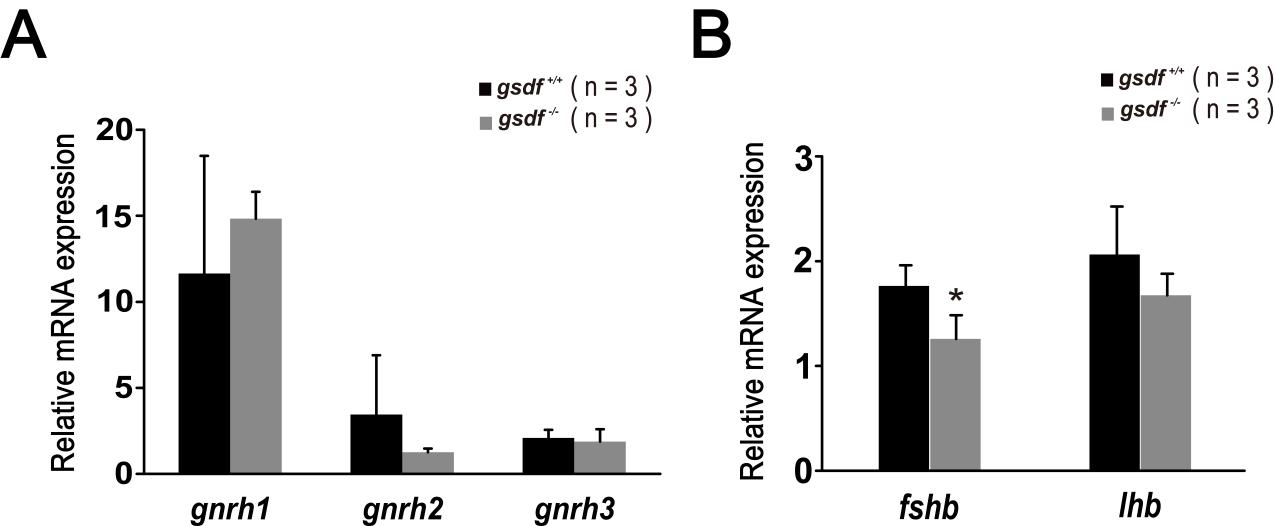


**Supplementary Figure 3**. Expression of hypothalamic and pituitary genes in XX *gsdf* ^+/+^ and *gsdf* ^-/-^ fish at 6 mah. (A) The hypothalamic *gnrh1*, *gnrh2* and *gnrh3* mRNA expression in XX *gsdf* ^-/-^ and *gsdf* ^+/+^ fish. (B) The pituitary *fshb* and *lhb* mRNA expression. *Fshb* was ignificantly down-regulated in XX *gsdf* ^-/-^ than *gsdf* ^+/+^ fish. Results are presented as mean ± SD. * *p* < 0.05.
